# A survey of facilitators and barriers to recruitment to the magnetic trial

**DOI:** 10.1186/1745-6215-14-S1-O60

**Published:** 2013-11-29

**Authors:** Geetinder Kaur, Rosalind Smyth, Colin Powell, Paula Williamson

**Affiliations:** 1 University of Liverpool, Liverpool, UK; 2 University College London (UCL), London, UK; 3 Children's Hospital for Wales, Cardiff, UK

## Background

Recruitment to randomised trials with children is challenging. It is imperative to understand the factors that boost or hinder recruitment to clinical trials. We conducted a survey of facilitators and barriers to recruitment to the MAGNETIC trial, using a web-based tool previously developed (1).

## Method

MAGNETIC is a multi-centre randomised trial of nebulised magnesium in acute severe asthma, recruiting over 500 children from 30 UK sites. Recruiters were asked to grade a list of factors from -3 to +3 depending on whether the factor was perceived as a strong, intermediate, or weak barrier (-3 to -1) or facilitator (+1 to+3) and (0) if thought to be not applicable. Free text responses were invited on strategies applied to counter the identified barriers and suggestions on future reorganisation of similar trials. We plan to look for correlation between responses and calibrated recruitment performance at sites.

## Results

The commonly identified facilitators were motivation and experience of study team, effective communication, presence of designated research nurses, good trial management, trial publicity and clarity in presentation of trial information. The commonly identified barriers were clinical workload, shift patterns, time and setting of consent, GCP training, non-availability of research staff out of hours and parents' concerns about their child taking an experimental medicine.

## Conclusions

This study highlights important generic and trial-specific facilitators and barriers to recruitment to a paediatric trial in the acute setting. This information can be very useful for successful conduct of future clinical trials with children.
